# Efficient Human Posture Recognition and Assessment in Visual Sensor Systems: An Experimental Study

**DOI:** 10.3390/s25216789

**Published:** 2025-11-06

**Authors:** Lei Lei, Haonan Zhang, Qi Zhang, Weihua Wu, Weijia Han, Runzi Liu

**Affiliations:** 1 School of Information Engineering, Xi’an University, Xi’an 710065, China; leilei@xawl.edu.cn; 2 School of Physics and Information Technology, Shaanxi Normal University, Xi’an 710119, China; zhanghaonan@snnu.edu.cn (H.Z.); wjhan@snnu.edu.cn (W.H.); 3 State Key Laboratory of ISN, School of Telecommunications Engineering, Xidian University, Xi’an 710071, China; dakexiaoqi@stu.xidian.edu.cn; 4 School of Information and Control Engineering, Xi’an University of Architecture and Technology, Xi’an 710055, China; rzliu@xauat.edu.cn

**Keywords:** visual sensor system, posture assessment system, deep learning, human posture recognition

## Abstract

Currently, recognition and assessment of human posture have become significant topics of interest, particularly through the use of visual sensor systems. These approaches can effectively address the drawbacks associated with traditional manual assessments, which include fatigue, variations in experience, and inconsistent judgment criteria. However, systems based on visual sensors encounter substantial implementation challenges when a large number of such sensors are used. To address these issues, we propose a human posture recognition and assessment system architecture, which comprises four distinct subsystems. Specifically, these subsystems include a Visual Sensor Subsystem (VSS), a Posture Assessment Subsystem (PAS), a Control-Display Subsystem, and a Storage Management Subsystem. Through the cooperation of subsystems, the architecture has achieved support for parallel data processing. Furthermore, the proposed architecture has been implemented by building an experimental testbed, which effectively verifies the rationality and feasibility of this architecture. In the experiments, the proposed architecture was evaluated by using pull-up and push-up exercises. The results demonstrate that the proposed architecture achieves an overall accuracy exceeding 96%, while exhibiting excellent real-time performance and scalability in different assessment scenarios.

## 1. Introduction

Regular physical exercise and maintaining fitness are paramount for preventing degenerative and cardiovascular diseases, providing substantial health advantages over a sedentary lifestyle [1]. This necessity has elevated exercises and physical fitness training to topics of widespread concern across various sectors, driven by the continuous improvement of residents’ living standards and the enhancement of national health awareness. For enterprises and institutions, the physical health of employees significantly impacts work efficiency and team collaboration [2]. In education institutions, as reforms in physical education curricula advance in primary and secondary schools, assessments of exercises and physical fitness have increasingly become important criteria for evaluating students’ overall quality [3]. This trend extends to higher education, where the assessment of fitness in colleges has garnered growing attentions from the Ministry of Education. As noted in the *Guiding Outline for Teaching Reform of “Physical Education and Health” (Tentative)* [4], the assessment of physical fitness for college students has become a crucial aspect of physical education. However, traditional assessment methods, which rely on manual observation, suffer from significant drawbacks such as subjectivity, rater fatigue, inconsistencies, and a lack of data traceability. To overcome these limitations, modern approaches have shifted towards sensor-based systems. In particular, the advent of deep learning and computer vision has provided powerful models for objective, automated posture assessment, offering significant advantages in accuracy, traceability, and efficiency over manual methods.

Recently, vision-based embedded sensor systems have become an increasingly popular solution for exercise assessment. These systems often draw from the broad field of Human Activity Recognition (HAR), which aims to automatically identify and classify human actions from sensor data. This field has diverse applications, including clinical analyses such as pathological gait recognition [5,6]. A fundamental component within this domain is Human Posture Recognition and Assessment (HPRA), a field that focuses on identifying spatial locations of a person’s joints and limbs to understand their body configuration and movement. In the context of exercise analytics, HPRA facilitates the real-time quantitative monitoring of body movements, offering an objective basis for performance evaluation [7]. However, systems with visual sensors face critical challenges regarding the efficiency of posture recognition and assessment, particularly when a large number of visual sensors are involved. In this case, their computational complexity often exceeds the capabilities of many low-spec computing platforms, such as the Phytium 2000+ [8]. Furthermore, the absence of a generalized algorithmic modeling framework results in significant performance limitations for visual sensor systems when assessing multiple exercise modalities, including but not limited to pull-ups, push-ups and abdominal crunches. Therefore, addressing these issues is a crucial step toward improving the intelligence and enabling the large-scale application of human posture recognition and assessment systems, thereby promoting their use in a wider range of scenarios to meet diverse assessment needs. It is important to note that such systems are not intended to replace the invaluable expertise of professionals like physiotherapists and kinesiologists. Rather, our vision-based system is explicitly positioned as a decisional support tool. It is designed to augment clinical and professional fitness evaluations by integrating expertise-derived assessment criteria with objective, quantitative, and traceable sensor data, thereby providing a powerful adjunct to overcome the subjectivity and inconsistency of purely manual methods.

To address these issues, this paper proposes a comprehensive and efficient approach for human posture recognition and assessment, along with an implementable architecture for a visual sensor system. Our results effectively tackle the challenges of low efficiency, poor accuracy, and limited scalability that are commonly associated with traditional assessment methods. The main contributions of this paper are summarized as follows:

- We present the design and practical implementation of an integrated four-subsystem architecture on a resource-constrained platform. We validate its effectiveness in concurrently processing data streams from multiple visual sensors, demonstrating its potential for managing the data loads characteristic of larger-scale scenarios and delivering assessment results in real-time.
- We demonstrate the successful application of a finite state machine (FSM) model to create a generalizable posture assessment framework. This framework is proven to be effective across different exercise modalities (pull-ups, push-ups, etc.) within our system, enhancing the fairness and transparency of the assessment process.
- We provide a comprehensive end-to-end validation of the entire system through a physical experimental testbed. The results confirm that our integrated solution achieves high accuracy over 96% and robust real-time performance, verifying its feasibility and practical value for deployment in real-world assessment scenarios.

## 2. Related Work

Human posture detection and action recognition technology have been widely applied in sports training, rehabilitation engineering, and intelligent healthcare. In sports training scenarios, accurate real-time recognition and counting of training actions are crucial for ensuring training quality and avoiding sports injuries. However, existing exercise assessment methods still face significant challenges.

The foundational method for exercise assessment is direct observation by a trained professional, such as a coach or clinician. In order to achieve exercise evaluation, manual methods often rely on human observers, which leads to inconsistency, lack of data traceability, and low efficiency in large-scale deployment [9,10]. To overcome these limitations, researchers first turned to sensor-based systems. Wearable devices [11,12,13], such as Inertial Measurement Units (IMUs) containing accelerometers and gyroscopes, can provide highly accurate data on limb orientation, velocity, and acceleration. While effective, early iterations of such systems sometimes required placing multiple sensors on the body, which could be intrusive. Furthermore, they occasionally suffered from issues like sensor drift and complex calibration procedures. It is important to note that modern advancements, such as e-textiles, have significantly mitigated the issue of intrusiveness, and newer IMUs often feature simplified, less time-demanding calibration processes [5,6]. Nonetheless, vision-based approaches offer a compelling non-contact alternative with the advent of deep learning.

State-of-the-art models for human pose estimation, such as OpenPose, HRNet, AlphaPose [14,15], and BlazePose [16,17,18,19,20,21], leverage deep neural networks to directly predict the 2D or 3D coordinates of key skeletal joints from an image or video frame [22,23,24]. Against this background, there is an urgent need to develop an action recognition and counting system suitable for sports training scenarios, which should integrate the advantages of computer vision to address the problems of poor real-time performance, ambiguous action standards, and insufficient robustness in existing technologies. Meanwhile, the system must not only enable simple, fast, and efficient deployment, but also judge the standardization of movements in accordance with sports physiology standards. Additionally, it should provide real-time feedback and accurate counting for users, thereby improving training effectiveness. In these models, although OpenPose, HRNet, and AlphaPose perform well in terms of recognition accuracy, they have limitations such as slow recognition speed and high requirements for hardware performance. In contrast, BlazePose is a model framework that combines fast recognition speed and high accuracy, with its core advantage being low hardware requirements. Our proposed human posture recognition and assessment system requires processing concurrent video streams from multiple cameras, and it is deployed on a Phytium 2000+ processor. These two factors, concurrent video processing demands and the hardware’s performance specifications, create constraints such that OpenPose, HRNet, and AlphaPose cannot be deployed on this platform, and only BlazePose can be effectively deployed.

Therefore, we finally choose BlazePose as the posture recognition model of the proposed system. It is worth noting that our research paradigm—focusing on system integration, deployment, and experimental validation on resource-constrained embedded or edge devices—is a widespread practice in the field of human pose analysis. For instance, the works by Huang et al. [25] focuse on implementing a lightweight OpenPose on edge devices like the Jetson TX2; the researches by Qian [26] and the work by Leone et al. [27] respectively demonstrate the complete engineering implementation of posture recognition on embedded systems using inertial sensors and machine learning. The core contribution of these studies is not the proposal of novel recognition algorithms, but rather the validation of a solution’s actual performance and operability in specific application scenarios (e.g., edge computing, wearable devices) through system design and experimentation. This study follows the same research philosophy, aiming to provide a practical and valuable system paradigm for the large-scale application of visual sensors on Low-Computing- Power platforms.

## 3. Experimental System Architecture

**Figure 1:**
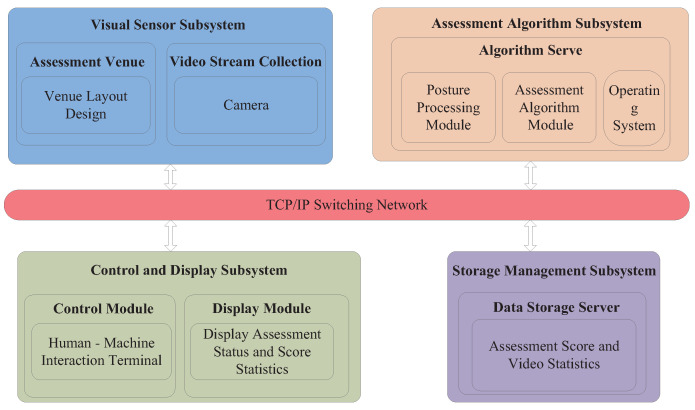
Framework diagram of human posture recognition and assessment system.

This section presents the architecture design of our testbed for the human posture recognition and assessment system. As depicted in Figure 1, the system architecture consists of four main subsystems. Each subsystem is responsible for distinct tasks and collaborates using the TCP/IP network protocol to facilitate data exchange. The four-subsystem architecture is deployed on the Phytium 2000+ processor platform. Tailored for Phytium 2000+, this design overcomes prior real-time pose estimation challenges on the hardware and enables simultaneous real-time assessment of multiple exercise modalities. Moreover, a state machine-based assessment algorithm is integrated into the assessment algorithm subsystem. Compared to traditional architectures, integrating state machines into the architecture to ensure accurate counting and scalability to other exercise modalities is a novel application.

### 3.1. Visual Sensor Subsystem

#### 3.1.1. Function Design

**Figure 2:**
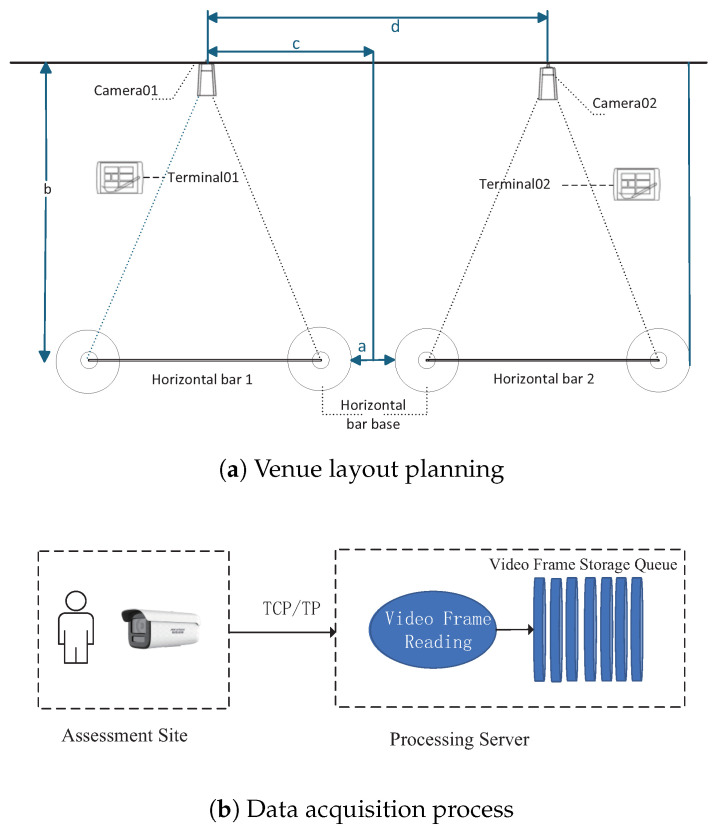
Venue layout and data acquisition process (Taking pull-ups as an example).

This subsystem includes venue layout planning and video stream acquisition, as illustrated in Figure 2a. Its primary objective is to ensure the real-time collection of video data through strategic venue arrangement, camera selection, and positioning. This framework is crucial for subsequent posture estimation and assessment. The data collection process entails the integration of software, hardware, and network coordination. Cameras capture video streams utilizing the Real-Time Streaming Protocol (RTSP), while OpenCV’s cv2.VideoCapture class facilitates the real-time reading of video frames from RTSP streams [28,29]. Each captured frame is stored in a video frame queue, enabling subsequent subsystems to access the raw data in a sequential manner. The effective selection of protocols, optimized queue management, and seamless hardware-software integration improve the capabilities of the subsystem regarding real-time performance, stability, and processing capacity. In video stream acquisition, since the assessment site and processing server are physically separated, data transmission between the camera and the server must traverse various networks. This scenario imposes the following core requirements on the camera:

**Real-time acquisition**: The camera must support low-latency video transmission to ensure that the system can capture and transmit images in real-time for posture recognition and scoring. Network transmission latency should be restricted to the millisecond range to guarantee prompt data delivery.

**Image quality**: High-resolution and clear video footage serve as the foundation for accurate posture recognition. The camera should support multiple resolution options to accommodate various assessment needs. By appropriately adjusting the resolution, the system can maintain image quality while optimizing network bandwidth, reducing storage requirements, and enhancing operational robustness.

**Video compression**: The adoption of efficient video compression technologies, such as H.264 and H.265, can significantly reduce the size of video data, enhance algorithm efficiency, and decrease network bandwidth consumption, as well as the amount of storage space used.

#### 3.1.2. Hardware Implementation

The selection of this specific camera over alternatives such as standard USB cameras constitutes a critical design decision, as it directly ensures the system’s scalability potential. A key rationale for this choice lies in the fact that network-based cameras are indispensable to a distributed architecture—a fundamental prerequisite for large-scale deployment across extended areas. Our system leverages a TCP/IP switching network to coordinate multiple devices even over potentially long distances, and this infrastructure aligns seamlessly with the capabilities of network cameras. Unlike USB cameras, which are limited by physical cable lengths and restricted to single-device connections, the network cameras in our design—equipped with an RJ45 port—can be effortlessly integrated into the distributed network. This integration not only enables the flexible deployment of numerous cameras to cover large or multiple assessment sites but also guarantees the stable transmission of video streams, thereby reinforcing the system’s ability to scale effectively [30]. The key technical specifications of the chosen camera are detailed in Table 1. The entire data collection process, illustrated in Figure 2b, involves collaborative coordination among software, hardware, and networking components. Each time a video frame is successfully obtained, it is stored in a video frame storage queue, enabling subsequent processing subsystems to read the raw data in sequence.

### 3.2. Assessment Algorithm Subsystem

#### 3.2.1. Function Design

This subsystem extracts human keypoint information from raw video data and executes the corresponding posture assessment algorithms to generate scores. The assessment algorithm subsystem includes a posture processing module and an assessment algorithm module.

**Figure 3:**
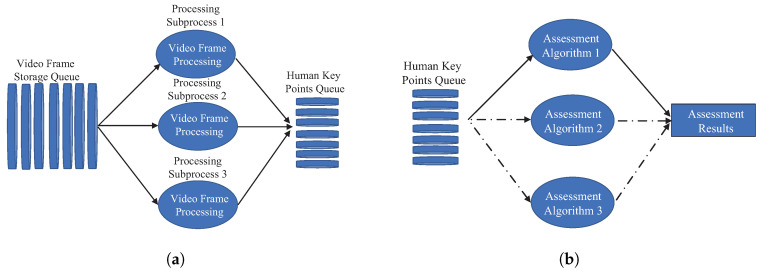
System processing subsystem. (**a**) Posture processing module. (**b**) Assessment algorithm module.

The posture processing module maintains two queues: one for storing raw video frames and another for processed keypoint coordinates. Both queues are thread-safe to prevent data contention or loss. Additionally, the system establishes appropriate queue sizes and regularly clears processed frames to optimize memory usage. As illustrated in Figure 3a, the posture processing module is responsible for acquiring video frames from the video frame storage queue, which is downstream of the visual sensor subsystem. It then utilizes relevant human posture detection algorithms to extract human keypoints from the video frames and transmits the core data of the keypoints to the subsequent human keypoint queue for the assessment algorithms to analyze, count and calculate the assessment scores. As depicted in Figure 3b, the assessment algorithm module obtains data from the human keypoint queue, which is downstream of the posture processing module. It automatically selects the corresponding assessment algorithm for processing based on the chosen assessment event, ultimately generating the assessment results.

#### 3.2.2. Hardware Implementation

In this system, posture processing and assessment are implemented on a server running the Kylin operating system [31], with the specifications detailed in Table 2. Kylin is a Linux kernel-based operating system designed for the Chinese market, offering high security and stability to support enterprise-level application operations. The system also requires a Python 3.8.0 environment and dependency libraries for algorithm execution, such as TensorFlow and OpenCV. A key design trade-off during hardware-software integration was the selection of the human posture recognition model, balancing accuracy and hardware compatibility. We evaluated four mainstream models: OpenPose, HRNet, AlphaPose, and BlazePose. OpenPose and HRNet deliver high accuracy but require GPU acceleration (e.g., NVIDIA Tesla V100) to process 1080P video at 15 FPS, which exceeds the computational capacity of the Phytium 2000+ processor. AlphaPose reduces GPU dependency but still requires 8+ CPU cores per video stream, limiting the system to 8 concurrent streams—insufficient for multi-station assessments. BlazePose, despite a slightly lower accuracy, achieves 25 FPS for 1080P video on the Phytium 2000+ processor and supports 16 concurrent streams. Experimental validation (Section 5.2 and Section 5.3) shows BlazePose meets the system’s accuracy requirements, making it the optimal choice for balancing performance, compatibility, and scalability.

### 3.3. Control and Display Subsystem

#### 3.3.1. Function Design

The control and display subsystem includes a control module and a display module. As the primary interface for user interaction, the control module is responsible for delivering user commands and ensuring timely responses from all subsystems to start the assessment process. The display module presents real-time algorithm execution and results, thereby enhancing transparency of the assessment process.

#### 3.3.2. Software Implementation

**Figure 4:**
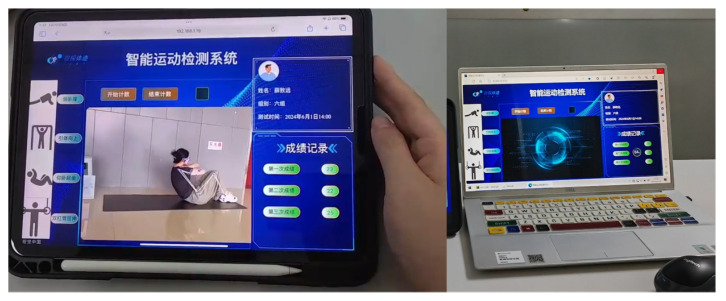
The UI of display module.

In this subsystem, we utilize Vue.js for the development of a control module and the user interface (UI) of the display module. Vue.js is a progressive frontend framework that simplifies UI development, enhances development flexibility, enables rapid response to user interactions, and thereby improves the overall UI development experience. The interaction between frontend and backend is achieved through Axios, a promise based HTTP client. This enables cross-domain access and resource requests to obtain real-time data and update the page. The user interaction component employs directives provided by Vue.js (such as v-bind and v-for) to bind data and update the page, ensuring a dynamic response to user commands and changes occurring on the page. The UI of display module, which runs on the tablet along with the tablet terminal, is illustrated in Figure 4.

### 3.4. Storage Management Subsystem

#### 3.4.1. Function Design

This subsystem is responsible for storing and managing all assessment data, including posture videos, scores, and related information. The data storage servers ensure secure storage and efficient retrieval of this data. In the storage management subsystem, video archives and assessment scores are primarily organized and stored in a database, which serves as the foundation for data analysis. After acquiring the assessment video stream, it is time-stamped according to the timestamps of the start and end frames. Subsequently, the video is archived in the folder designated for each test-taker, allowing for future querying and management.

#### 3.4.2. Software Implementation

**Figure 5:**
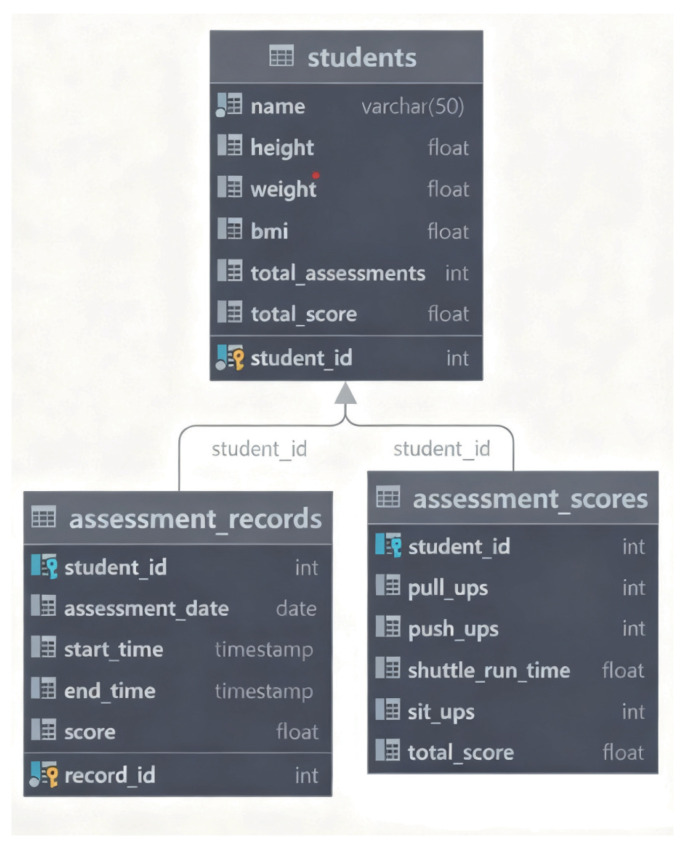
Table structures and their relationships in databases.

The system utilizes the widely-used MySQL database for efficient management. Within this database, a primary schema named DATABASE has been established, and several tables have been created within it. These tables include a student table (In the table, “students” refers to the test-takers undergoing posture assessment.), an assessment records table and an assessment scores table. The key relationships among these tables are illustrated in Figure 5.

The students’ table serves as a primary data store for the essential information of each student. This information includes the student’s name, height, weight, BMI (Body Mass Index), total number of assessments, total score, and more. This table provides a comprehensive profile for each student, enabling the system to monitor their physical condition and assessment history. This, in turn, facilitates the subsequent analysis and management of their health status and changes in physical fitness.

The assessment records table captures essential information for each assessment, including the assessment date, start and end times, as well as the score for each assessment. By designing this table, the system can accurately record the timing and results of each assessment activity, enabling precise tracking of the specific details of each assessment in subsequent analyses.

The assessment scores table primarily records the specific scores for each evaluation, including scores for various exercise modalities such as pull-ups and push-ups. Each score is associated with an individual student and a corresponding assessment record, thereby ensuring high accuracy and integrity of the assessment data. This table not only captures each student’s performance across different modalities but also serves as foundational data for the comprehensive scoring and progress tracking of the system.

Through the close association of these three tables, the database can effectively and comprehensively manage students’ personal information, assessment records and score data. The scores from each assessment are linked to the students’ basic information and historical records, facilitating detailed analysis and queries by the users. It is important to note that both the assessment_records and assessment_scores tables are linked to the students table through the student_id key. This direct relationship allows for comprehensive queries on a per-student basis and makes an additional associative key between the records and scores tables unnecessary.

## 4. Algorithms of Human Posture Recognition and Assessment

The algorithmic core of our system extends beyond static keypoint detection to include comprehensive posture modeling and assessment. Posture reconstruction employs BlazePose model to generate anatomical landmarks [32,33,34], which are then refined using temporal smoothing. From the reconstructed posture sequences, we extract joint angles and segment distances within a standardized 2D image coordinate system, allowing for precise geometry-based assessment. Posture-specific assessment logic is implemented as a FSM, where each state corresponds to a recognized phase of posture (e.g., high-pull, low-support). Transitions between states are triggered when posture metrics meet predefined thresholds. This approach ensures that only complete and accurate motion cycles contribute to the final score.

### 4.1. Smoothing and Filtering

**Figure 6:**
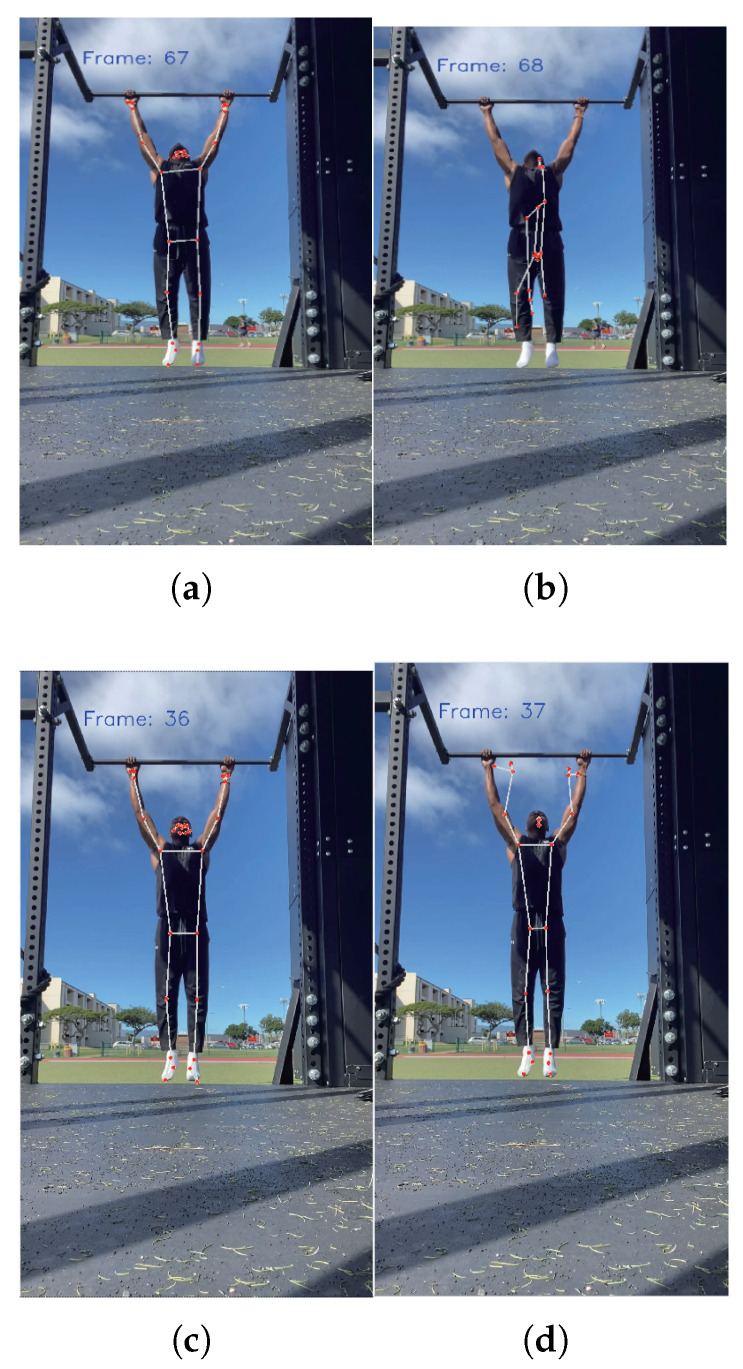
Coordinate errors in recognition. (**a**) Frame-67 posture is correctly recognized. (**b**) Frame-68: Pose Recognition Flying Point. (**c**) Frame-36 posture is correctly recognized. (**d**) Frame-37 posture recognition jitter.

Figure 6 demonstrates the continuous frame outputs of human keypoints at a video frame rate of 30 FPS. As shown in Figure 6a, the keypoint detection in the 67th frame accurately reflects the current human posture. However, in Figure 6b, although the human body does not undergo significant postural changes in the 68th frame, erroneous keypoint detection occurs. Some points show excessive deviations from their actual joint positions, and “flying points” appear in the image. Similarly, the keypoint detection in the 36th frame shown in Figure 6c is accurate; however, in the 37th frame shown in Figure 6d, the detected position of the hand exhibits minor jitter.

To optimize the “flying points” and coordinate jitter errors in human keypoints detection, we employ a frame-based inter-frame joint correction and smoothing algorithm. This algorithm effectively enhances the keypoints detection results by implementing inter-frame position correction and sliding window smoothing.

The pseudocode of the algorithm presented in Algorithm 1 briefly illustrates the execution process of the algorithm. Special attention should be given to the configuration of the correction threshold and the size of the smoothing window. For the correction threshold, the thresholds for *x*-axis and *y*-axis are set to 1/30th of the frame width and height, respectively. This determination is based on experimental test results. The size of the sliding window for the smoothing process is set to 10 frames.
**Algorithm 1** Frame-Sequence-Based Joint Correction and Smoothing Algorithm**Require:** 
Original keypoint frame sequence *K* (dimension: (number of frames, keypoint data), where keypoint data includes (x,y)), correction threshold *T*, smoothing window size *W***Ensure:** 
Corrected and smoothed keypoint frame sequence SK  1:**Initialization:** Set correction threshold *T*; Set smoothing window size *W*  2:**for** i→K **do**  3:   Compare the (x,y) dimensions of keypoint data between current frame *i* and previous frame i−1. If the difference exceeds *T*, correct *i*’s keypoint position to align with i−1.  4:**end for**  5:**for** i→CorrectedK (denoted as C_K) **do**  6:   For (x,y) of frame *i* in C_K, compute the average within a window of size *W* centered at *i*, and replace the original coordinates.  7:**end for**

### 4.2. Body Angle Measurement

**Figure 7:**
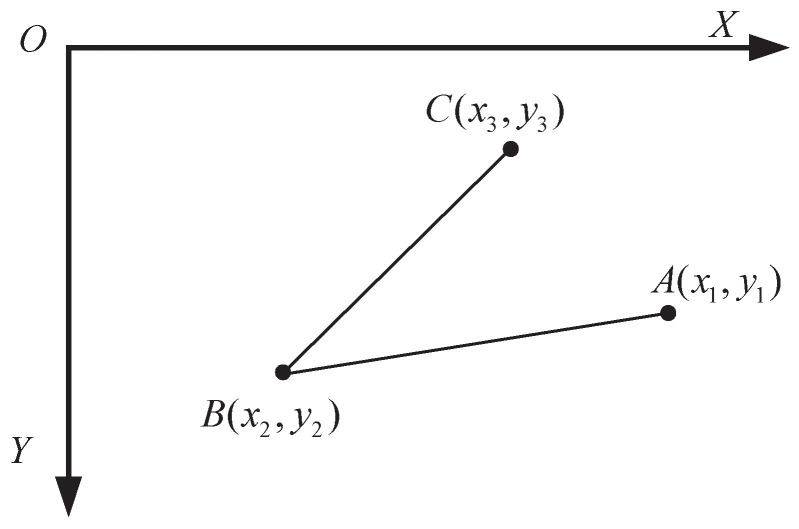
2D screen image coordinate system XOY.

In the analysis of assessment algorithms, the angles between joints and the distances between keypoints are crucial for assessing human posture [35,36,37]. Therefore, prior to implementing the algorithm, it is necessary to standardize these angles and distances. It is important to note that the raw data consists of normalized scale factors that range from (0,1). To facilitate practical image processing and calculations, these factors are multiplied by the actual width and height of the image frame to obtain pixel coordinates. Based on these coordinates, a 2D image coordinate system (XOY) is established, as shown in Figure 7.

In the coordinate system (XOY), the origin *O* is located at the top-left corner of the image. The *x*-axis extends horizontally to the right, while the *y*-axis extends vertically downward. Given three human keypoints A,B, and *C*, the angle ∠ABC between skeletal vectors BA and BC can be calculated through the following steps [38]

(1) $$BA=(x_{1}-x_{2},y_{1}-y_{2}),$$

(2) $$BA=\sqrt{{\left(x_{1}-x_{2}\right)}^{2}+{\left(y_{1}-y_{2}\right)}^{2}},$$

(3) $$BC=(x_{3}-x_{2},y_{3}-y_{2}),$$

(4) $$BC=\sqrt{{\left(x_{3}-x_{2}\right)}^{2}+{\left(y_{3}-y_{2}\right)}^{2}}.$$

The cosine value of the joint bone radian is defined as follows:

(5) $$\cos\left(\theta \right)=\frac{BA\cdot BC}{\left|BA||BC\right|}.$$

Subsequently, the radian value is obtained using the arc-cosine formula, with the counterclockwise direction from the origin of the coordinate system XOY defined as positive. For improved intuitive understanding, this radian value is then converted into degrees. In the two-dimensional image plane, the distance *d* between keypoints, such as joint points *A* and *B*, can be expressed as:

(6) $$d=\sqrt{{\left(x_{2}-x_{1}\right)}^{2}+{\left(y_{2}-y_{1}\right)}^{2}}.$$

Note that the distance *d* is measured in pixels on the 2D image frame.

### 4.3. Pull-Up Assessment Algorithm Design

#### 4.3.1. Horizontal Bar Height Measurement

Since the system employs a purely vision-based solution without prior camera calibration, the height of the horizontal bar must be estimated during the execution of the algorithm. By analyzing the entire pull-up movement, we observe that after the test-taker completes the upward motion to grasp the bar, a distinct feature emerges: the height of the hands remains constant. Based on this observation, we attempt to design an algorithm to identify a stable hand height value. Variance measures data dispersion and reflects the extent of value fluctuations within a dataset. The formula for calculation is as follows:

(7) $$\mathrm{Var}\left(X\right)=\frac{1}{n}\sum_{i=1}^{n}{\left(x_{i}-\mu \right)}^{2},$$

where $X=\{x_{1},x_{2},\ldots ,x_{n}\}$ represents the dataset, μ is the mean of the dataset, *n* is the number of elements in the dataset, and $x_{i}$ denotes each data point within the dataset.

By calculating the variance, the algorithm can effectively identify regions where the heights remain highly stable and change minimally. During the pull-up process, when the hands are in a stationary or nearly static position, the height variation of the hands is minimal, resulting in a low variance value. Conversely, when the hands move up and down, the height variation increases, leading to a rise in variance. By observing these phenomena, the algorithm can accurately determine the height value when the hands are stable, which is then used as the height of the horizontal bar. This approach helps avoid errors in height estimation that could arise from unstable hand movements. The specific steps for the algorithm to estimate the height of the horizontal bar are as follows:

**Calculate the variance within the sliding window**: The sliding window method is employed, and the variance is calculated for each window. Given a window size ω, for the data in each window [i,i+ω], calculate its variance:

(8) $$\mathrm{Var}\left(X\right)=\frac{1}{\omega }\sum_{j=i}^{i+\omega }{\left(x_{j}-\mu \right)}^{2},$$

where μ denotes the mean value of the data within the window, $x_{i}$ represents each data point, and ω signifies the size of the window.

**Variance stability detection**: Select windows that have relatively small variance values, specifically:

(9) $$\mathrm{Var}\left(X_{i:i+\omega }\right)<threshold,$$

where threshold is a threshold determined by the algorithm’s configuration, representing the acceptable range of variance.

**Filter and select the maximum height value**: Among all stable intervals, the algorithm selects the one with the highest average height value as the final horizontal bar height:

(10) $$H_{\max}=\max\left(H_{1},H_{2},\ldots ,H_{n}\right),$$

where $H_{i}$ represents the average height value of the *i*-th window, and *n* denotes the total number of window intervals that meet the variance stability condition.

**Output result**: Return the value of the height of the horizontal bar $H_{\max}$.

#### 4.3.2. Exercise Posture Measurement Based on Finite State Machine

A FSM is a model that describes how a system switches between a finite number of states in accordance with inputs and based on fixed rules. In exercise posture assessment, the state machine can define the postures occurring at each stage of a movement. It evaluates whether the movement meets the standard state transition conditions by utilizing human keypoint data, thereby facilitating automated scoring. Based on the different movement states that may occur during the pull-up process of the human body, the states of the state machine model in this paper are defined as follows:

**State1:**  The state in which the human body is pulled to a high position during a pull-up.
**State2:**  The state in which the human body exits State1 during a pull-up.
**State3:**  The state in which the human body exits State4 during a pull-up.
**State4:**  The state in which the human body is in a low position during a pull-up.

The transition between each state on the state machine is closely related to the output of the state machine judgment function F(), which is presented in Algorithm 2.

**Figure 8:**
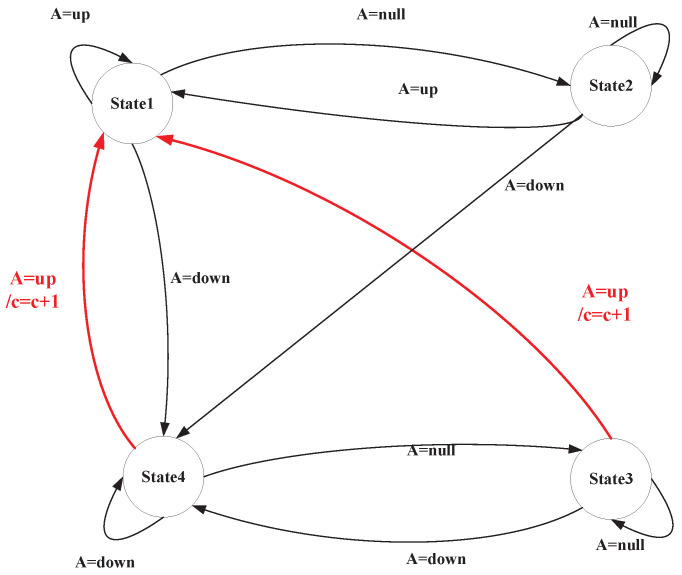
Pull-up assessment algorithm state machine.

This paper presents a state transition table (Table 3) and a state transition diagram (Figure 8) for the pull-up assessment algorithm. The following sections provide a detailed examination of each state and its transition conditions. This will facilitate a deeper analysis of the behavioral patterns and assessment criteria associated with pull-up movements.
**Algorithm 2** Pull-up State Machine Judgment Function F()**Require:** 
Mouth height, Horizontal bar height $H_{\max}$, Elbow height, Eye height, Angle of the human elbow, Elbow angle threshold**Ensure:** 
Posture  1:**if** Mouth height $>H_{\max}$ **then**  2:   Posture =“up”  3:**else if** Eye height < Elbow height & Angle of the human elbow > Elbow angle threshold**then**  4:   Posture =“down”  5:**else if then**  6:   Posture =“null”  7:**end if**

**Transitions from State1**: In State1, the system remains in this state only when the output of the judgment function F() is “up” indicating that the human body is in the high-pull position. When the output of function F() is “null”, it indicates that the human body exits the “up” state and transitions to State2. When the output of function F() is “Down ”, the FSM transitions to State4. This indicates that the human body is moving directly from the high position to the low position. This generally suggests that the test taker’s movement speed may be too fast, or that the sampling frequency of posture detection is inadequate.

**Transitions from State2**: State2 represents the state that slides out from State1. When the output of the judgment function F() is “null”, FSM system remains in State2. If the output of function F() is “up” the system re-enters State1 (the high position). This repetition is not counted because the human body does not transition through State4 (the low position). When the output of function F() is “down”, the FSM proceeds to State4, where the human body descends to the low position.

**Transitions from State3**: State3 represents the state that slides out from State4. When the output of the judgment function F() is “null”, the FSM remains in State3. If the output of function F() is “up”, the FSM transitions to State1 (the high-pull position) and records this as a standard pull-up posture that has been completed. When the output of function F() is “down”, the FSM re-enters State4 (the low position).

**Transitions from State4**: State4 indicates that the human body is in a low position during pull-ups. When the output of the judgment function F() is “down”, the FSM remains in State4. When the output of the function F() is “up”, the FSM transitions to State1 (the high position) and counts this as a completed standard pull-up posture. This may also indicate that the test taker’s movement speed is too fast or that the sampling frequency for posture detection is insufficient. When the output of the function F() is “null”, the FSM exits State4 and transitions to State3.

This state machine model clearly defines key states, such as “pull position”, “transition states” and “low position” along with their transition conditions. It can identify standard postures, where only the complete process from State4 to State1 is counted, and effectively handle abnormal situations (such as the direct transition from State1 to State4 due to fast movements). The strict conditional constraints in the state transition table ensure the rigor of the counting logic, thereby avoiding misjudgments and omissions. The intuitive presentation of the state transition diagram makes the behavioral patterns of the entire assessment process clearly distinguishable. This state machine-based design method meets the stringent requirements of exercise posture assessments, and also demonstrates good fault tolerance and real-time performance.

### 4.4. Push-Up Assessment Algorithm Design

To accurately describe the working principle of the state machine, we define the relevant terms involved in the push-up assessment algorithm to enhance understanding of its mechanism. As shown in Table 4, the term “angle” refers to the joint angle calculated in the 2D XOY coordinate system of images. The term “height” is defined as the relative distance between the maximum *y*-axis value in the image frame and the *y*-value of the key point positions. Lastly, “threshold” refers to an empirical threshold set according to assessment standards, or it may be a self-determined threshold based on each individual’s performance during exercise.

Based on the different movement states that the human body may experience during push-ups, we have defined the various states of the state machine as follows.

**State1:**  During push ups, the body supports a high position.
**State2:**  The state in which the human body exits from State1 during push ups.
**State3:**  The state in which the human body exits from State4 during push ups.
**State4:**  The body supports a low position during push ups.
**State5:**  The posture of the human body in a misaligned position during push ups.

The transition between various states in the state machine is closely related to the output of the state machine judgment function F(), the pseudocode of which is presented in Algorithm 3.
**Algorithm 3** Push-up State Machine Judgment Function F()**Require:** 
Elbow Ang., Mouth Height, Hip Ang., Torso Ang., Elbow Exten. Thresh., Mouth Down Thresh., Body Exten. Thresh., Torso Ang. Thresh.**Ensure:** 
Posture  1:**if** Hip Ang. < Body Exten. Thresh. **then**  2:   Posture =“error”  3:   **return** “error”  4:**end if**  5:**if** Elbow Ang. > Elbow Exten. Thresh. **then**  6:   Posture =“up”  7:**else if** Mouth Height < Mouth Down Thresh. & Torso Ang. < Torso Ang. Thresh.**then**  8:   Posture =“down”  9:**else**10:   Posture =“null”11:**end if**

**Figure 9:**
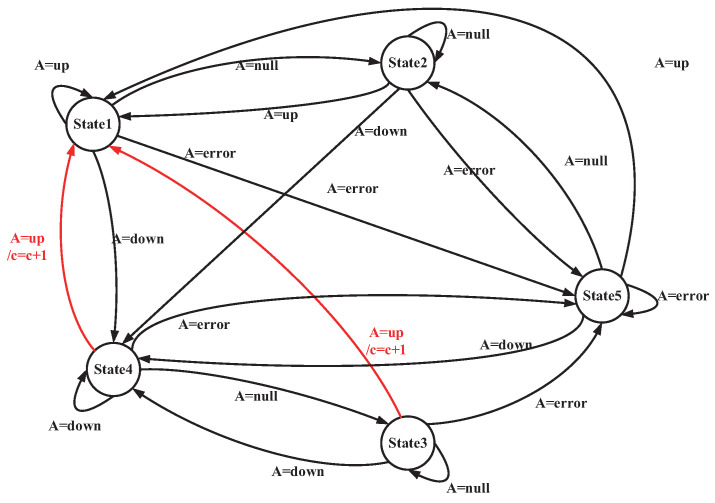
Push-up assessment algorithm state machine.

To clearly illustrate the working principle of the push-up state machine, this paper provides a state transition diagram and a state transition table as shown in Table 5 and Figure 9. The principle of state machine transition for push-up is essentially the same as that for pull-up. To save space, it will not be repeated here.

### 4.5. Sit-Up Assessment Algorithm Design

Based on the different movement states that the human body may experience during sit-up, we have defined the various states of the state machine as follows.

**State1:**  When leaning up, the distance between the elbow and knee is less than the threshold value, i.e., Sit-up-threshold.
**State2:**  The state in which the human body exits from State1 during push ups.
**State3:**  The state in which the human body exits from State4 during push ups.
**State4:**  When lying down, the angle between the body and the horizontal line is less than the threshold value, i.e., Sit-down-threshold.
**State5:**  The posture of the human body in a misaligned position during sit-ups.

The transition between various states in the state machine is closely related to the output of the state machine judgment function F(), the pseudocode of which is presented in Algorithm 4.
**Algorithm 4** Sit-up State Machine Judgment Function F()**Require:** 
Distance between elbow and knee, Angle between the body and the horizontal line, Sit-up-threshold, Sit-down-threshold**Ensure:** 
Posture  1:**if** The distance between the elbow and knee is less than Sit-up-threshold **then**  2:   Posture =“up”  3:   **return** “error”  4:**end if**  5:**if** The angle between the body and the horizontal line is lower than Sit-down-threshold**then**  6:   Posture =“down”  7:**else**  8:   Posture =“null”  9:**end if**

The state transition diagram and a state transition table are same as Table 5 and Figure 9, respectively. The principle of state machine transition for sit-up is essentially the same as that for pull-up. To save space, it will not be repeated here. Moreover, it is evident that the state machine-based posture assessment method proposed in this paper exhibits strong generalizability and extensibility. The core idea lies in defining key motion states and transition rules to establish a standardized assessment framework. This framework does not rely on the specific features of movements, but rather focuses on the universal patterns of human motion. Whether it is upper limb-dominant movements such as push-ups and pull-ups, or full-body coordinated movements like squats and sit-ups, this framework can be used for exercise assessment. Consequently, this state machine-based approach not only fulfills the assessment requirements for specific exercises in the current study, but also provides a scalable technical pathway for future applications in diverse exercises (e.g., track and field, gymnastics). Its strong adaptability underscores its practical value for broader implementation.

## 5. Experiments and Tests

In this section, we present the experimental studies conducted by using the aforementioned testbed. It should be noted that all participants were provided with the same set of instructions prior to data acquisition. They were asked to perform each exercise to their maximum capacity while adhering to standard athletic form. For pull-ups, the standard was defined as starting from a full-hang position with arms extended and pulling up until the chin cleared the horizontal bar. For push-ups, the standard required maintaining a straight torso while lowering the body until the chest was near the floor, followed by a full extension of the arms. This protocol was intentionally designed to test the robustness of the assessment algorithm against the natural performance variations inherent in a diverse population.

### 5.1. Experimental Configuration

To debug and optimize the algorithm, the following outlines the workflow of human posture recognition and assessment task. The Human Pose Detection (Pose Landmarker [34]) task utilizes the create_from_options function to initialize the task, while the create_from_options function accepts the configuration option values that need to be processed. Table 6 presents the parameter configurations along with their descriptions.

The Pose Landmarker task exhibits distinct operational logic across its various execution modes. In IMAGE or VIDEO mode, the task blocks the current thread until it has fully processed the input image or frame. Conversely, in LIVE STREAM mode, the task invokes a result callback with the detection outcomes immediately after processing each frame. If the detection function is called while the task is occupied with other frames, it simply ignores the new input frame. In this system, although the live stream from the webcam inherently aligns with the LIVE STREAM mode, the VIDEO mode is deliberately chosen to ensure detection accuracy and fairness in assessment calculations. This decision benefits from the multi-process design of the assessment algorithm subsystems. Even when the posture processing speed lags behind the data collection speed, this design guarantees that all image frames are processed. Additionally, the parallel operation of multiple sub-modules within the assessment algorithm subsystem plays a crucial role in maintaining real-time performance.

Each time the Pose Landmarker task executes a detection, it returns a Pose LandmarkerResult object. This object contains the coordinates of each human body keypoint, providing essential data for subsequent analysis and assessment.

### 5.2. Pull-Up Case

**Figure 10:**
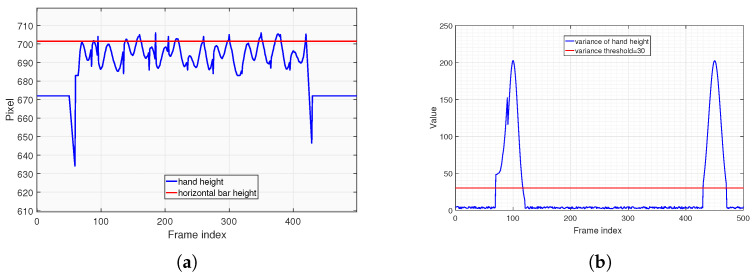
Horizontal bar height measurement of pull-up. (**a**) Hand height across video frames during pull-ups. (**b**) Variance of hand height values within each sliding window.

In Figure 10a, the blue line represents the changes of hand height across video frames during pull-ups. It can be observed that the hand height changes slightly and remains stable during the initial and final stages. This stability occurs because the tester’s hands do not vary significantly when standing either before starting or after finishing the pull-up assessment. However, during the pull-up process, there are slight jitters and variations about hand coordinates, primarily influenced by minor hand movements and errors of body keypoints recognition. Particularly during the initial stage of pull-ups, the hand height experiences significant changes. The blue line in Figure 10b illustrates the variance of hand height values within each sliding window. It is evident that the variance remains small in frame intervals where the hands do not move significantly. The red line denotes the acceptable variance threshold (30 pixels, an empirical value selected based on the resolution of the subject’s video stream). Within the sliding window intervals that meet this variance threshold, we select the average hand height value from Figure 10a and take its maximum value (as indicated by the red line) as the height of horizontal bar.

**Figure 11:**
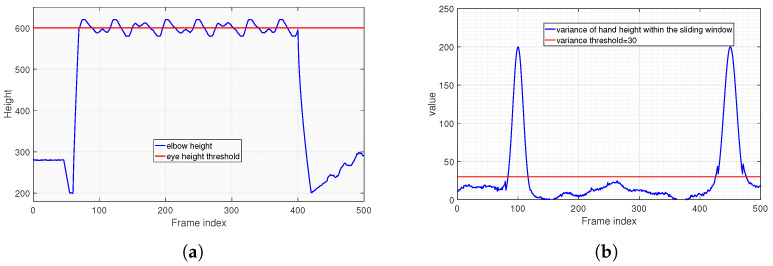
Eye height threshold measurement of pull-up. (**a**) Elbow height across video frames during pull-ups. (**b**) Variance of elbow height values within each sliding window.

Similarly, the algorithm employs the same deductive logic to determine the eye height threshold, which will be used by the state machine judgment function F() in Algorithm 2. Throughout the entire pull-up process, the elbow joint point displays similar data characteristics with the hand height in Figure 10. The algorithm extracts the stable height value after the tester mounts the bar during the assessment. This value acts as the threshold that the eyes should remain below when descending to the low position of pull-up, which is referred to as the eye height threshold. The algorithm test results are presented in Figure 11, and the deduction process will not be reiterated here.

**Figure 12:**
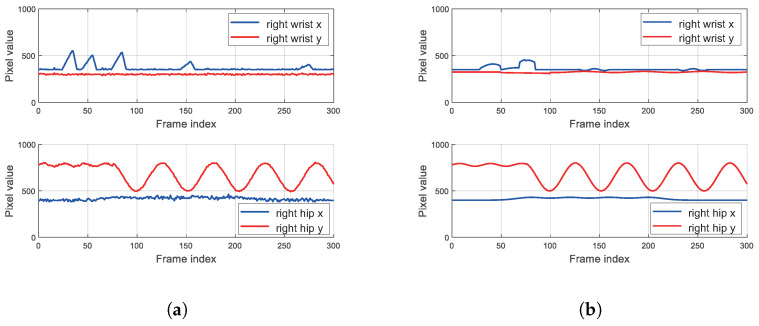
Data results of applying the frame-sequence-based joint correction and smoothing algorithm. (**a**) Data results after the first-step correction. (**b**) Final data results after further smoothing.

Figure 12 presents the results of applying the frame-sequence-based joint correction and smoothing algorithm. To clearly demonstrate the execution process of the algorithm, the data results after the first-step correction are shown in Figure 12a, while the final results after further smoothing following the correction are presented in Figure 12b. It can be observed from Figure 12a that the errors caused by outliers are effectively suppressed after the correction. In Figure 12b, after the step of smoothing, the jitter errors of the coordinates are also successfully eliminated. This demonstrates that the algorithm effectively optimizes the errors in the recognition process and restores the true motion trajectory, thus providing a solid foundation for the design of the subsequent assessment algorithm.

In the preceding sections, the assessment criteria for pull-ups have been analyzed in detail, and a corresponding algorithm has been developed based on these criteria. To verify the actual effectiveness of the designed algorithm, we conduct the following experimental validations.

**Figure 13:**
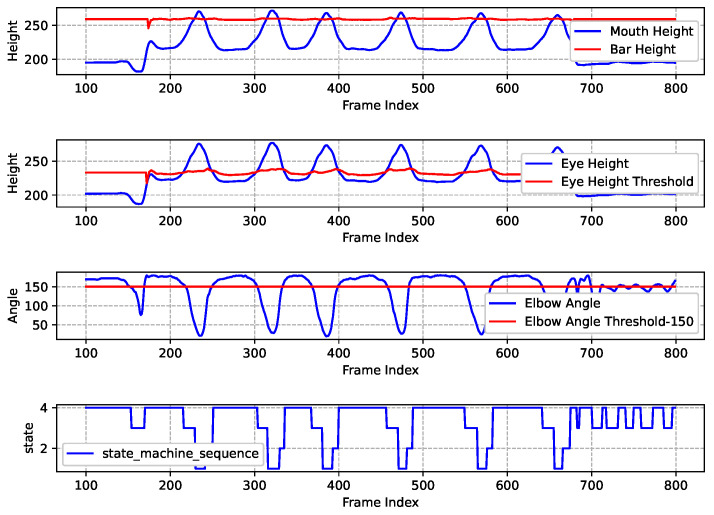
Standard pull-up exercise recognition and assessment results.

The data results graph from the standard pull-up experiment is shown in Figure 13. The figure is divided into four sub-graphs from top to bottom, illustrating the variation of each key data point. The analysis of the experimental data is as follows.

**The 1st Sub-graph**: The blue line illustrates the height of the human mouth as the sequence of frames progresses, while the red line indicates the height of the horizontal bar as estimated by the horizontal bar height measurement algorithm. It is intuitively apparent that during a standard pull-up exercise, the curve representing the mouth’s height consistently intersects with the straight line denoting the height of the horizontal bar.

**The 2nd Sub-graph**: The blue line represents the variation of eye height throughout the frame sequence, while the red line indicates the estimated eye height threshold by Figure 11. Similarly, the curve depicting eye height consistently intersects with the straight line representing the eye height threshold.

**The 3rd Sub-graph**: The blue line illustrates the variation of the angle of human elbow as the frame sequence progresses, while the red line represents the elbow angle threshold, which has been set at 150 degrees (an empirical value). This setting encourages the test-taker to keep their elbows fully extended when lowering to the low position.

**The 4th Sub-graph**: The blue line illustrates how the state machine varies as the frame sequence changes, allowing for observation of the overall state of human exercise and the logical counting process. Based on the sequence of the state machine in the 4th sub-graph, this experiment concludes that the test-taker performed 6 standard pull-up movements. Consequently, there were 6 transitions from State4 to State3 to State1 in the sequence.

**Figure 14:**
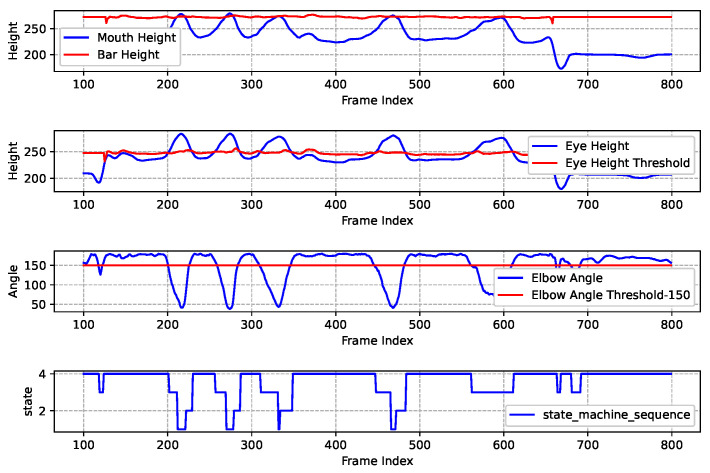
Nonstandard pull-up exercise recognition and assessment results.

Figure 14 shows the results of a pull-up assessment process that encounters multiple errors. The data indicates that only a few movements meets the standard assessment criteria. The main issues identified include: the mouth height not exceeding the horizontal bar during the upward pull and the eyes not being below the designated threshold height. Consequently, the final assessment score is determined to be 4, which corresponds to 4 sequences of “State4-State3-State1” in the state machine.

Then, we organized various test-takers to test the pull-up assessment algorithm, and the results are shown in Table 7. To evaluate the recognition accuracy of the pull-up algorithm, a set of experimental trials was conducted involving multiple participants performing pull-ups. During these trials, the actual number of valid pull-up repetitions performed by each participant was meticulously recorded as ground truth by experienced human assessors. The recognition accuracy of the system for pull-ups was then calculated by comparing the number of repetitions correctly identified by our algorithm against this manually established ground truth. Specifically, the accuracy represents the ratio of correctly recognized repetitions to the total actual repetitions, expressed as a percentage, thus quantifying the ability of the system to precisely count valid movements. Specifically, the accuracy rate shown in the table is calculated as the ratio of correctly recognized repetitions by the algorithm to the total actual repetitions established by human assessors, expressed as a percentage. Overall, the assessment algorithm demonstrates a relatively high accuracy rate among test-takers with high physical fitness levels. However, the recognition accuracy rate decreases slightly among ordinary college students and teachers. The overall accuracy rate of the algorithm is 96.6%, which indicates that the algorithm can effectively assess pull-ups in most cases.

### 5.3. Push-Up Case

**Figure 15:**
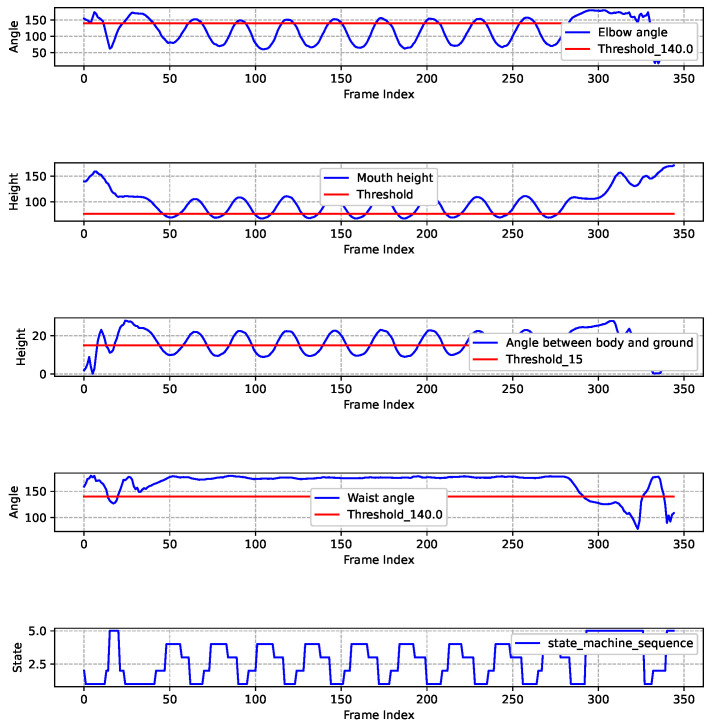
Standard push-up exercise recognition and assessment results.

Figure 15 presents the results of a standard push-up experiment. It includes five subgraphs arranged from top to bottom, illustrating the changes of multiple key data points. The specific analysis is as follows.

**The 1st Sub-graph**: The blue line illustrates the variations of elbow angle of human body as the frame sequence progresses. The red line represents the elbow angle threshold which is set as 140 degrees. This empirically chosen value aims to prevent excessive bending of the elbow during the upward phase. This threshold can be adjusted at different actual conditions. When the blue line is above the red line, it indicates that the elbow angle meets the criteria for the high support position; otherwise, it does not fulfill the established standard.

**The 2nd Sub-graph**: The blue line illustrates the variation of mouth height, indicating the position of the tester’s mouth relative to the ground during push-ups. The mouth height fluctuates throughout the movement. The red line represents the minimum shoulder height throughout the entire push-up process, which can be different for different individuals. A necessary condition for entering the low support state during push-ups is that the mouth height must be lower than the minimum shoulder height.

**The 3rd Sub-graph**: The blue line illustrates the variation of the angle between torso and ground, while the red line indicates a threshold of 15 degrees.

**The 4th Sub-graph**: The blue line illustrates the variation of waist angle. The red line indicates the threshold which is set at 140 degrees. The purpose of this threshold is to ensure that the tester maintains a straight body.

**The 5th Sub-graph**: The blue line illustrates the changes of the state machine sequence. When the blue line of the state machine experiences a transition from State4 to State3 to State1, it signifies that the tester has successfully completed a standard push-up movement. During the movement, any non-compliant action will trigger State5. Finally, it can be observed from the figure that the result of this push-up assessment is 9.

**Figure 16:**
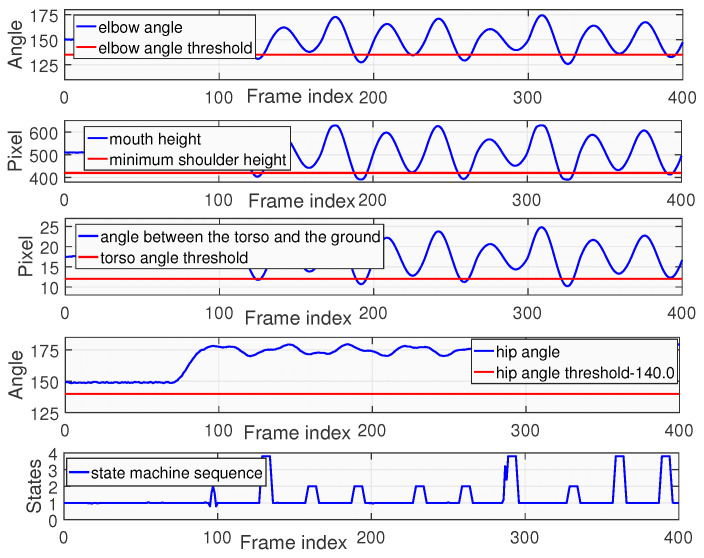
Nonstandard push-up exercise recognition and assessment results.

Figure 16 shows the assessment results of non-standard push-up. In this experiment, the mouth height consistently failed to reach to the required threshold for the low-position state, and the torso angle also did not meet the standard. Therefore, in the state machine sequence, only 4 transitions from State4 to State3 to State1 happen. Thus, the final score of this tester is 4.

Similarly, we organized various test-takers to test the push-up assessment algorithm, and the results are shown in Table 8.

### 5.4. Sit-Up Case

**Figure 17:**
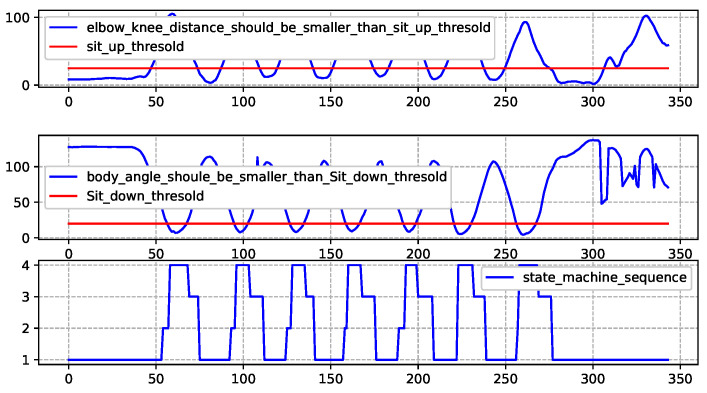
Standard sit-up exercise recognition and assessment results.

Figure 17 shows the different stages of the sit-up exercise.

**The 1st Sub-graph**: The blue line illustrates the distance between elbow and knee. The red line represents the sit-up-threshold. This threshold can be adjusted at different actual conditions. When the blue line is above the red line, it indicates that the the elbow and knee are in contact with each other.

**The 2nd Sub-graph**: The blue line illustrates angle between the body and the horizontal line. The red line represents the sit-down-threshold. When the blue line is above the red line, it indicates that the human body is in a lying state.

**The 3rd Sub-graph**: The blue line illustrates how the state machine varies as the frame sequence changes, allowing for observation of the overall state of human exercise and the logical counting process. Based on the sequence of the state machine in the 4th sub-graph, this experiment concludes that the test-taker performed 7 standard sit-up movements.

In the aforementioned pull-up, push-up, and sit-up experiments, we conducted tests with individual subjects. In fact, our system is designed under the specification that one camera is dedicated to capturing one person to ensure accurate posture recognition. When there are multiple people for testing simultaneously, we recommend configuring multiple cameras (one camera per person) to avoid interference between individuals. When occlusions occur or lighting conditions are poor, the posture processing module may inaccurately detect key points, leading to the phenomenon of “flying points”. In this case, the Joint Correction and Smoothing Algorithm can identify and correct abnormal key point positions caused by occlusions or bad lighting to a certain extent, thereby improving the stability and accuracy of posture recognition.

### 5.5. Computational Efficiency Analysis

**Figure 18:**
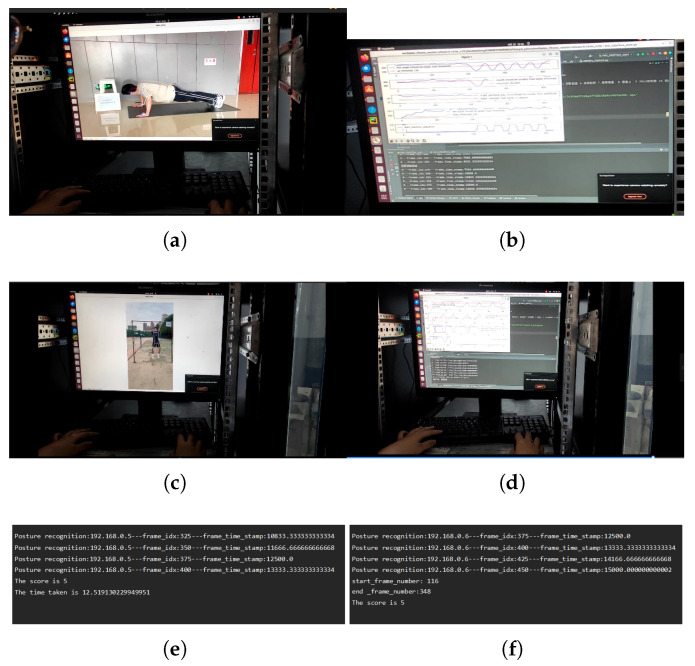
Data Processing Process and Real-Time Computational Performance. (**a**) The server processes push-up videos; (**b**) Algorithm Analysis of Push-ups; (**c**) The server processes pull-up videos (**d**) Algorithm Analsysis of Pull-Ups (**e**) Push-up assessment judgment results (**f**) Pull-up assessment judgment results.

To provide a clearer picture of the operational performance of the system, we conducted the computational efficiency analysis. Figure 18 illustrates the server’s data processing flow during the test of both push-up and pull-up assessments. For debugging and demonstration purposes, the live video feed and real-time algorithm analysis are displayed, as depicted from Figure 18a–d. These display features are disabled during actual deployment to minimize resource consumption.

The assessment results are shown in the final panels of Figure 18e,f. Figure 18e illustrates the processing of a push-up assessment, where 5 valid repetitions were completed in 12.52 s from stream initiation to final calculation. Figure 18f shows a similar pull-up assessment of 5 repetitions taking 13.17 s. In both cases, the calculated scores are returned to the user interface within 3 s of the tester completing the exercise. This performance demonstrates the capability of the system for real-time analysis and rapid feedback, meeting the requirements for efficient and timely sports assessment.

### 5.6. Discussion

Our developed system offers a comprehensive approach to automated exercise assessment, distinguishing itself by integrating multiple functionalities into a robust, end-to-end solution suitable for practical deployment—with explicit consideration of fault handling and design trade-offs to address real-world constraints. While much of the existing state-of-the-art research often focuses on optimizing individual components, such as advanced pose estimation algorithms or novel deep learning models for specific exercise recognition, our system prioritizes the entire workflow from real-time video capture to score calculation and data management—including targeted handling of practical faults inherent in visual sensor-based assessment. The primary non-quantifiable faults in this context are “flying points” and coordinate jitter of human keypoints, caused by mathematically unmodelable environmental interference like uneven lighting and limb occlusion. Instead of relying on theoretical fault quantification, we adopted an engineering validation approach: building an experimental testbed based on the Phytium 2000+ processor, collecting real motion data from 5 test groups, and implementing a frame-based inter-frame joint correction and Smoothing Algorithm. As shown in Figure 12, this algorithm reduces “flying point” errors to ensure the reliability of subsequent posture assessment—a practical fault mitigation strategy that aligns with the system’s real-world deployment goal, where experimental validation of fault handling is more actionable than theoretical quantification.

This integrated design is crucial for real-world scenarios where not just accuracy, but also reliability, interpretability, and ease of deployment are paramount. The choice of a modular architecture and a rule-based FSM for exercise evaluation represents a deliberate design decision. Unlike purely data-driven, black-box deep learning approaches— which can achieve high accuracy but often lack transparency—our FSM model provides clear, human-interpretable rules for what constitutes a valid repetition. While our system is designed to be robust, extreme conditions could still pose challenges. Specifically, our calibration-free design, while enhancing deployment flexibility, makes the 2D-based assessment potentially vulnerable to significant alterations in viewing perspective or large-scale camera displacements. The lack of a calibration phase to compensate for such geometric variations is a limitation of the current study. Additionally, while our smoothing algorithm mitigates partial occlusions, more serious or prolonged occlusions can still lead to keypoint detection failures, which may cause the FSM to register errors. Finally, as the system relies on fixed visual sensors, it has limited portability for assessments in unconstrained environments compared to wearable devices like IMUs [6]. Furthermore, our methodological choice to allow subjects to perform exercises ’freely’ to their maximum capacity, while reflective of a real-world scenario, introduces performance variability that might affect results. This approach relies heavily on the FSM’s robustness to filter non-standard movements, and its impact versus a strictly protocol-driven execution should be noted as a study limitation. Rigorous experimental testing validated the system’s efficacy, achieving 96.6% accuracy for pull-ups and 97.4% for push-ups, confirming its alignment with expert judgment. In summary, our system offers a robust, accurate, and transparent solution for automated sports assessment, effectively bridging the gap between advanced computer vision research and practical application. Furthermore we plan to integrate additional thresholds, such as elbow angle checks, alongside the existing eye and mouth criteria, to further minimize the likelihood of false positives in our assessment logic.

## 6. Conclusions

This paper presented the design and implementation of an automated human posture recognition and assessment system, developed to overcome the issues of traditional manual methods. A primary contribution is our novel evaluation pipeline, which uses a temporal correction algorithm to ensure reliable movement trajectories and a FSM model to enforce strict, phase-based logic, accurately counting valid repetitions while rejecting non-standard movements. Future iterations might explore multi-camera setups or integration with other sensor modalities to further enhance robustness against extreme environmental factors. We also plan to integrate 3D human pose estimation models, which would not only enhance robustness but also unlock more sophisticated biomechanical analyses. Ultimately, this work delivers a high-fidelity, scalable solution for automated exercise assessment, with significant potential for deployment in educational, military, and enterprise contexts where fair and consistent evaluation is paramount.

## Figures and Tables

**Table 1:** Camera Technical Specifications [30].

Parameter	Description
Resolution	Maximum 6 megapixels (3072×2048 Ultra HD). Main stream supports 1920×1080, 1280×720; Sub-stream supports 1280×720, 640×480.
Wide Dynamic Range (WDR)	120dB WDR for clear images in high-contrast lighting environments.
Lens Type	Multiple focal lengths available (2.8 mm, 4 mm, 6 mm) for flexible installation.
Video Coding	Supports H.264, H.265, H.265+ compression formats to reduce bandwidth and storage requirements.
Frame Rate	25FPS@3072×2048. Triple-stream technology supports simultaneous output of main, sub, and tertiary streams.
Network Protocols	Supports RTSP and other real-time streaming protocols.
Interface	Standard RJ45 Gigabit Ethernet port for stable wired connection.

**Table 2:** List of Identification Processing Server Configurations [31].

No.	Component Configuration
1	Phytium 2000+ Processor, Main Frequency 2.2 GHz, Cores 64 Memory: DDR4 3200 32 G × 2, Supports 8 Memory Slots
2	Maximum Supports 38 Hot-Swappable Hard Drives 4 TB 3.5 × 7.2 K 6 Gb SATA Drive × 3/480 G 2.5 SATA 6 Gb R SSD × 2/Rear O2 Disk Drive
3	Supports 6 PCIe Expansion Slots, Board-Integrated Dual 120-Gigabit Electrical Ports Supports Wake-on-LAN, Network Redundancy, Load Balancing and Other Advanced Network Features
4	1 GB SAS 12 GB 8 RAID Card Supports RAID 1, 5, 6, 10, 50, 60
5	1 R45 Management Interface, 1 VGA Interface, 6 USB Interfaces Integrates BMC Chip, Supports IPMI2.0, SOL, KVM Over IP, Virtual Media and Other Advanced Management Functions
6	800W Power Socket × 2 Infrared 150 cm National Power Supply

**Table 3:** Pull-up Assessment Algorithm State Transition Table.

$S^{n}$	$S^{n+1}/Y$		
	Up	Down	Null
State1	State1/0	State2/0	State2/0
State2	State1/0	State4/0	State2/0
ine State3	State1/1	State4/0	State3/0
ine State4	State1/1	State4/0	State3/0

**Table 4:** Definition of Key Terms for Push-up Assessment.

Term	Specific Definition
**Joint Angles**	
Torso Ang.	Angle of shoulder → foot → hand joints (2D XOY coordinate system).
Hip Ang.	Angle of foot → hip → shoulder joints.
Elbow Ang.	Angle of shoulder → elbow → hand joints.
**Height Params**	
Mouth Height	Δ Y between max *y*-axis in frame and mouth’s y-coordinate.
**Thresholds**	
Mouth Down Thresh.	Minimum height of shoulders during the project process.
Elbow Exten. Thresh.	Min angle required at push-up’s highest position.
Body Exten. Thresh.	Min angle to maintain during the push-up motion.
Torso Ang. Thresh.	Max torso-ground angle allowed in push-up’s low-position state.

**Table 5:** Push-up Assessment Algorithm State Transition Table.

$S^{n}$	$S^{n+1}/Y$			
	Up	Down	Error	Null
State1	State1/0	State4/0	State5/0	State2/0
State2	State1/0	State4/0	State5/0	State2/0
ine State3	State1/1	State4/0	State5/0	State3/0
ine State4	State1/1	State4/0	State5/0	State3/0
ine State5	State1/0	State4/0	State5/0	State2/0

**Table 6:** Pose Landmarker Parameter Configuration Sheet.

Option Name	Description	Parameter Value Range
running_mode	IMAGE: For single image input mode. VIDEO: For video parsing mode. LIVE_STREAM: For real-time mode with input video stream (e.g., from a camera).	{IMAGE, VIDEO, LIVE_STREAM}
min_pose _detection_confidence	The minimum confidence value for successful pose detection.	Float [0.0, 1.0]
min_tracking_confidence	The minimum confidence value for successful pose tracking.	Float [0.0, 1.0]
output_segmentation_masks	Modify the returned pose segmentation masks.	Boolean
result_callback	Pose result callback, for receiving and processing results in real-time when the user selects the real-time stream mode.	ResultListener

**Table 7:** Pull-up Assessment Algorithm Accuracy Statistics.

Tester	Actual Repetitions/Rep	Detected Repetitions/Rep	Algorithm Counting Accuracy Rate /%
Ordinary Student	18	16	88.9
Teacher	22	21	95.5
PE Student 1	30	29	96.7
PE Student 2	35	34	97.1
Soldier	40	40	100
Summary	145	140	96.6

**Table 8:** Push-up Assessment Algorithm Accuracy Statistics.

Tester	Actual Repetitions/Rep	Detected Repetitions/Rep	Algorithm Counting Accuracy Rate /%
Ordinary Student	23	21	91.0
Teacher	34	33	97.0
PE Student 1	40	39	97.5
PE Student 2	45	44	97.8
Soldier	50	50	100
Summary	192	187	97.4

## Data Availability

The original contributions presented in this study are included in the article. Further inquiries can be directed to the corresponding author.
